# Hippocampal Hypertrophy and Sleep Apnea: A Role for the Ischemic Preconditioning?

**DOI:** 10.1371/journal.pone.0083173

**Published:** 2013-12-13

**Authors:** Ivana Rosenzweig, Matthew J. Kempton, William R. Crum, Martin Glasser, Milan Milosevic, Sandor Beniczky, Douglas R. Corfield, Steven C. Williams, Mary J. Morrell

**Affiliations:** 1 Department of Neuroimaging, Institute of Psychiatry, King's College, London, United Kingdom; 2 Danish Epilepsy Centre, Dianalund, Denmark; 3 Academic Unit of Sleep and Breathing, National Heart and Lung Institute, Imperial College London, London, United Kingdom; 4 NIHR Respiratory Disease Biomedical Research Unit at the Royal Brompton and Harefield NHS Foundation Trust and Imperial College London, London, United Kingdom; 5 Department for Environmental and Occupational Health, University of Zagreb, School of Medicine, Andrija Štampar School of Public Health, Zagreb, Croatia; 6 Department of Clinical Neurophysiology, Aarhus University Hospital, Aarhus, Denmark; 7 Manchester Medical School, University of Manchester, Manchester, United Kingdom; University of Naples Federico II, Italy

## Abstract

The full impact of multisystem disease such as obstructive sleep apnoea (OSA) on regions of the central nervous system is debated, as the subsequent neurocognitive sequelae are unclear. Several preclinical studies suggest that its purported major culprits, intermittent hypoxia and sleep fragmentation, can differentially affect adult hippocampal neurogenesis. Although the prospective biphasic nature of chronic intermittent hypoxia in animal models of OSA has been acknowledged, so far the evidence for increased ‘compensatory’ neurogenesis in humans is uncertain. In a cross-sectional study of 32 patients with mixed severity OSA and 32 non-apnoeic matched controls inferential analysis showed bilateral enlargement of hippocampi in the OSA group. Conversely, a trend for smaller thalami in the OSA group was noted. Furthermore, aberrant connectivity between the hippocampus and the cerebellum in the OSA group was also suggested by the correlation analysis. The role for the ischemia/hypoxia preconditioning in the neuropathology of OSA is herein indicated, with possible further reaching clinical implications.

## Introduction

Obstructive sleep apnoea (OSA) is a highly prevalent multisystem disease that affects up to 30-40% of selected patient populations [1-4] and presents an independent risk factor for stroke[5-7]. It also likely exacerbates the stroke damage, as well as increases the risk of a subsequent stroke[3,8]. OSA is predicted to become an even greater health problem in the future because two of its most prominent risk factors, obesity and older age, are on the rise[2,3]. Patients with OSA suffer repeated nocturnal episodes of pharyngeal obstruction, resulting in the intermittent hypoxia (IH), reoxygenation, episodic arousals and sleep fragmentation[9,10]. Recent studies also suggest that the elderly patients with OSA co-morbidity may suffer accelerated brain atrophy, cognitive decline and the onset and severity of dementia[1,3,9,11]. Notwithstanding this, OSA-associated brain injury is commonly reported as subtle[12], its associated neurocognitive deficits as mild and diffuse, and their full or partial reversibility by the current gold standard treatment continuous positive pressure airway (CPAP), as debatable[9,10,12-18]. 

The root of this discrepancy has been previously attributed to the use of different image analysis methods in various studies over the years, varied statistical thresholds and lack of OSA-standardised battery of sensitive neurocognitive tests[12]. This explanation, however, disregards the inter-individual heterogeneity to a given hypoxic stimulus during OSA[19] and it likely also discounts for the effects of sleep staging on regional neuronal vulnerability to episodic arousal and oxidative stress[10,20]. Equally, we consider that it does not account for any cardiovascular and cerebrovascular protection conferred by ischemic preconditioning, resulting from the nocturnal cycles of hypoxia-reoxygenation[10,19]. Ischemic preconditioning represents a generalized adaptation to ischemia by variety of cells that was initially demonstrated in the cardiovascular system of patients with OSA and later shown to occur in several other organs, including brain[19,21]. In OSA, during the apnea the activation of several gene programs (e.g. including the hypoxia inducible factor-1)[22] is thought to induce vascular remodelling, neo-angiogenesis, productive autophagy, reactive gliosis, various synaptic alterations, and to increase adult neurogenesis[4,19,23-26].

**Figure 1:**
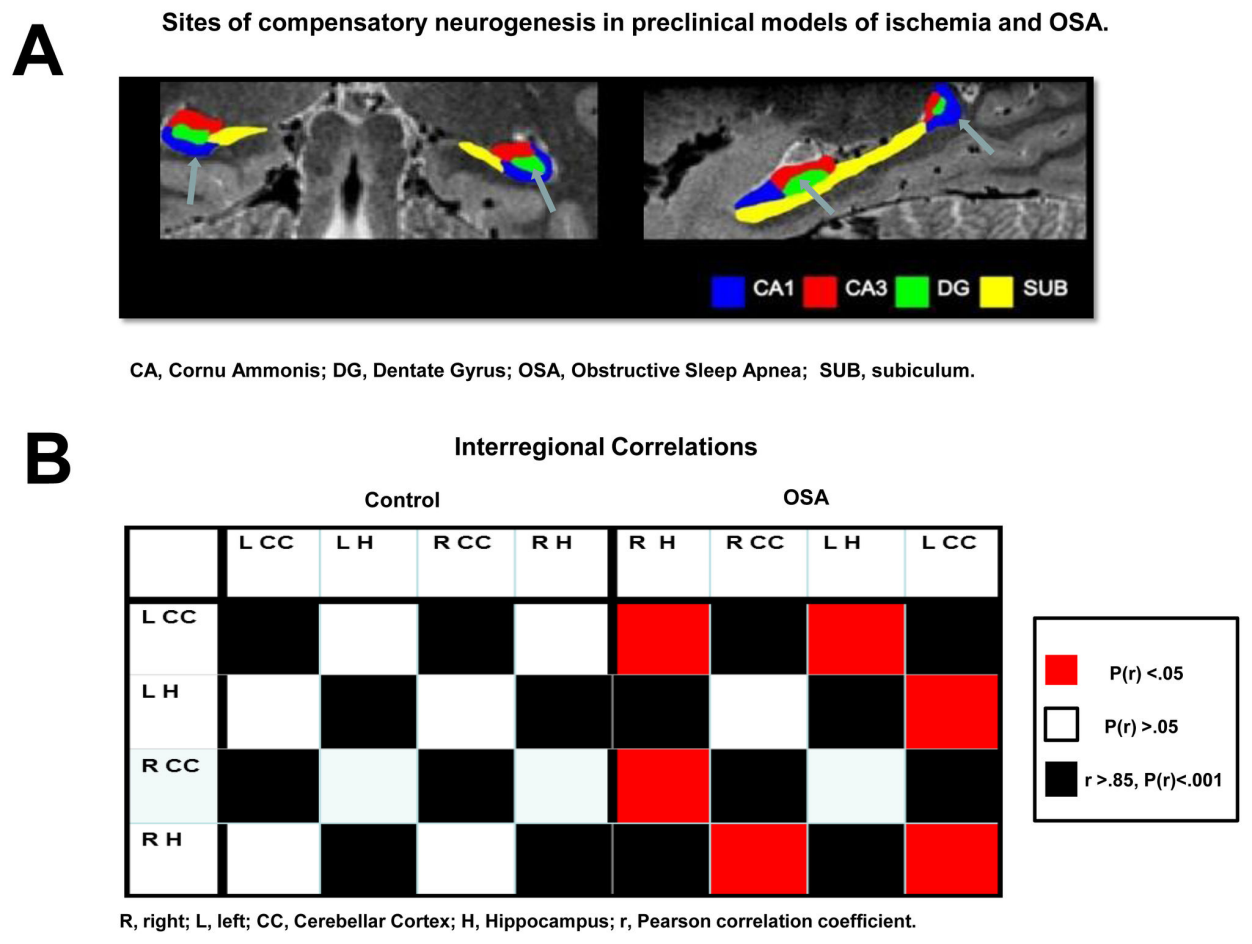
Sites of compensatory neurogenesis in preclinical models of ischemia and OSA. **A**) Dentate gyrus and CA1(arrows) support neurogenesis in animal models of ischemia and OSA [29,31,33]; human hippocampal subfields are shown (coronal/sagittal planes), adapted with permission from [72]. **B**) Potential aberrant connectivity between the hippocampus and cerebellum in OSA patients.

New neurons are produced on a continuous basis in normal adult human brain well into senescence with neural stem/progenitor cells residing in two major neurogenic regions: the subventricular zone lining the lateral ventricles and the dentate gyrus (DG) of the hippocampal formation[27,28]. Hypoxic/ischaemic insults in rodent models are powerful stimulators of adult neurogenesis in both neurogenic niches, and otherwise dormant regions such as the striatum and hippocampal pyramidal cell layer CA1 (Figure **1A**)[29]. Chronic IH in animal models of OSA is associated with impaired spatial learning that coincides with the increased apoptosis in the cortex and CA1 region of the hippocampus [4,30,31]. Gozal and colleagues demonstrated increased proliferation in the DG at a later stage of this process, which was present despite of the ongoing noxa. It was suggested that biphasic, temporal change in DG proliferation may account for the partial recovery of clinical function in the later stages of IH exposure[31]. In accord, several other preclinical studies demonstrated protective nature of moderate IH suggesting that ischemic preconditioning-like processes may occur[4,32]. For example, in one rodent model, the IH intervention after the ischemic event lead to increased expression of brain derived neurotrophic factor (BDNF), increased hippocampal neurogenesis and functional synaptogenesis, as well as in improvement in spatial learning and long-term memory impairment[24,25]. In another study, IH in adult rats was also shown to promote hippocampal neurogenesis, and to mimic antidepressant-like effects[33]. Recently, IH protocols have been also investigated as a tool to “prime” neural progenitor cells prior to transplantation into the injured CNS[34]. 

Despite the abundance of animal data suggesting the association between chronic intermittent hypoxia, ischemic conditioning and the subsequent adaptive increase in adult neurogenesis in several affected brain regions, this putative association has so far proved elusive in clinical studies of the CNS changes in OSA[12,14,35-40]. To date, volumetric, predominantly voxel based morphology (VBM), studies of CNS changes in OSA patients, including those performed by our group, point to predominantly hypotrophic effects in number of cortical regions and subcortical structures[14,35-38,40,41]. However, there is high variability in results across clinical studies of OSA and the findings are not always concordant between different neuroimaging methods[12,42]. Moreover, many of the neuroimaging methods used in earlier studies might not be sufficiently sensitive to authoritatively record subtle and spatially diffuse changes in regions such as hippocampal formation and the (cerebello)-thalamocortical oscillator. The connectivity between these regions is considered by some to present the core neurocircuitry of OSA neuropathology[10,43]. 

In order to address some of these issues, we used magnetic resonance imaging and the fully automated volumetric analysis method, FreeSurfer (FS), to study changes in several subcortical structures in mixed severity OSA patients and age-matched healthy controls[44]. The FS method has been extensively validated in a number of clinical studies where it was shown efficient in quantifying subcortical volumes in dementia[45], epilepsy[46] depressive disorders[47] and aging[48]. Our *a priori* hypothesis was that volumes of hippocampus, thalamus and cerebellum would differ across diagnostic groups. 

## Methods

Patients were recruited from Royal Brompton and Charing Cross Hospitals sleep clinics. Inclusion criteria were apnoea/hypoponea index (AHI) >15 events/h. Exclusion criteria were a history of respiratory disease, cerebrovascular/ischaemic heart disease, diabetes mellitus, neurological/psychiatric disorder, alcohol or illicit drug abuse, or current intake of psychoactive medications.

The same exclusion criteria were used for controls who were recruited from a database of healthy volunteers. Additionally, those with a history of sleep problems or habitual snoring were excluded. Polysomnography and questionnaires were used to exclude OSA (AHI <5 events/h); apneas were defined as >80% drop in airflow for 10s and hypopneas were defined as >50% reduction in airflow from baseline with a >4% dip in saturation, or an arousal from sleep. The study was part of an on-going research programme to investigate the impact of OSA on the brain; some images were previously assessed as a sub-set of a wider study [14]; all subjects gave informed written consent. 

### 2.1 Ethics

This study was approved by the Brompton Harefield & NHLI Research Ethics Committee. Written informed consent was obtained from each participant and the scans were anonymously analyzed. All clinical investigations were conducted according to the principles expressed in the Declaration of Helsinki.

### 2.2 Magnetic resonance imaging and image analysis

All participants underwent MR imaging and T1-weighted MR images were acquired using a 1.5T scanner (Magnetom Vision, Siemens Healthcare, Camberley, Surrey, UK) and a 3D MP-RAGE sequence (TI 300 ms, TE 4 ms, in-plane resolution 1.0x1.0 mm) with contiguous 2 mm coronal slices.

The T1-weighted images were processed and volumetry performed using an automated method, FreeSurfer, as previously described[44,45,47-50]. During this fully automated process removal of non-brain tissue, automated Talairach transformation, segmentation of the subcortical white matter (inclusive of segmentation of corpus callosum to five parts)[51] and deep grey-matter volumetric structures, intensity normalization, and cortical reconstruction were done. A neuroanatomical label was assigned to every voxel in the MR image volume, where the probability of a label at a given voxel was computed not just in terms of the grey-scale intensities and prior probabilities at that voxel, but also as a function of the labels in a neighbourhood of the voxel in question. Given our *a priori* hypothesis regarding the differences in hippocampal volumes, this step was particularly pertinent as it enabled correct separation of the hippocampus and amygdala, which have similar grey-scale values[44]. The analysis was performed using parallel running streams with no variability to the data processing conditions[52]. The segmented 3D images of structures of interest were inspected for gross errors through visualization with 3D slicer (Version 3.2 1.0, NIH, USA) (IR), and the volume values were extracted by implemented Unix scripts (WC and MK).

The Kolmogorov-Smirnov test was used to test the normality of distributions. To analyse differences in variety of demographic parameters between controls and OSA patients, Student’s t-test was initially applied (Table **1**). All statistical analyses had a 2-tailed α level of <.05 for defining significance and were performed by a biostatistician (MM) on the statistical software “STATISTICA 10.0” (http://www.statsoft.com). The gender differences between the two groups were found non-significant (Pearson Chi-Square test, P=.450). The intracranial volume (ICV) calculated by the FreeSurfer did not differ significantly between groups (t-test, P=.514) and a one-way analysis of covariance (ANCOVA) was conducted (age as a covariance) on the ICV normalised data (i.e. volume/ICV) to assess between-group differences (Table **2**). Finally, we also explored the presumed interregional connectivity between the hippocampi and cerebellar cortices with Pearson correlations; controlled for ICV and age[53]. 

**Table 1:** Demographic Information.

	**OSA** n= 32; mean [SD]	**Control** n= 32; mean [SD]
**Age (years)**	48.50 [12.51]	49.91 [11.43]
BMI (kg/m^2^)*	31.48 [4.34]	24.94 [3.61]
AHI (events/h)*	42.3 [23.81]	2.1 [1.61]
ODI (events/h)*	31.4 [19.43]	1.2 [1.33]
ESS*	13.2 [4.64]	4.7 [3.69]
**Right-handedness (%)**	100	100

* Significant difference between OSA patients and healthy controls (P<.001). There was no significant difference in the age between the two groups (P=.64). Normality was checked using Kolmogorov-Smirnov test. The plots appeared approximately normally distributed so independent sample *t-test* statistics were used to compare patients and controls.

Abbreviations: **AHI**, apnoea/hypopnoea index; **BMI**, body mass index; **ESS**, Epworth sleepiness scale; **n**, number; **ODI**, oxygen desaturation index; **OSA**, obstructive sleep apnoea; **SD**, standard deviation.

**Table 2:** Subcortical Volumes as determined by FreeSurfer.

**Structure**	**Controls** (n=32) mean [SD]	**OSA** (n=32) mean [SD]	**ANCOVA** ^a^ *P* values	***t* – test** ^a^ *P* values
***Right Hippocampus***	4168 [502]	4337 [462]	.049*	.042*
Left Hippocampus	4301 [475]	4454 [477]	.067	.057
Right Thalamus	7055 [1054]	6719 [827]	.094	.225
Left Thalamus	7196 [1059]	6966 [961]	.302	.539
Right Cerebellum (Cortex)	54105 [5345]	52464 [4974]	.416	.495
Left Cerebellum (Cortex)	52681 [5146]	51001 [5119]	.366	.459
Right Cerebellum (White Matter)	15310 [2491]	14659 [1777]	.363	.411
Left Cerebellum (White Matter)	15116 [2414]	14772 [1985]	.758	.852

In the table, for each neuroanatomical structure statistical analysis of group differences for volumes normalised to the ICV was performed; t-test and ANCOVA test (covariate with age), were done. Volumes are given as mm^3^.

^a^Bonferroni corrected P values. *Significant difference between OSA patients and healthy controls (P<.05). **Abbreviations**: **ANCOVA**, Analysis of covariance; **ICV**, intracranial volume; **OSA**, obstructive sleep apnoea; **SD**, standard deviation.

## Results

Sixty four participants were studied with MR neuroimaging (Table **2**). *A priori* hypothesis investigation concentrated on analysis of group differences for three neuroanatomical structures previously shown as affected in OSA, hippocampus, thalamus and cerebellum. The hippocampi were found larger bilaterally in OSA group and increase on the right was statistically significant (absolute mean values OSA, 4336.5 mm^3^ vs control, 4167.7 mm^3^). A statistically non-significant trend for smaller thalami in the OSA group was noted, more so on the right (absolute mean values OSA, 6718.9 mm^3^ vs control, 7054.6 mm^3^). No statistically significant differences were noted between cerebellar cortical and white matter volumes of the two studied groups (Table **2**). 

Amongst the values for several other FreeSurfer automatically calculated subcortical structures, only two more group differences in volumes reached statistical significance; those of choroid plexus and the middle anterior portion of corpus callosum. Both left (F (1,62)=4.08, P=.048) and right choroid plexus (F (1,62)=5.36, P=.024) were found hypertrophic in OSA group (Table **S1**). Conversely, the volume of the mid-anterior portion of the corpus callosum was significantly decreased in OSA patients (F (1,62)=4.47, P=.039). The results for the remaining subcortical structures calculated by FS are summarized in the Table **S1** **.**

Post hoc interregional correlation analyses (Figure **1B**; Table **S2**) revealed positive correlations in the OSA group (n=32) for both hippocampi with dominant cerebellar cortex (right, r=0.379; P=.032; left, r=0.357; P=.045) and for the right hippocampus with ipsilateral cerebellar cortex (r=0.363; P=.041). None of these correlations were significant in the control group (n=32).

## Discussion

During OSA, changes in cerebral blood flow occur[54] and apnea-induced hypoxemia combined with reduced cerebral perfusion likely predisposes patients to nocturnal cerebral ischemia[55,56], as well as hypoperfusion of certain brain regions during the awake states[57]. The evolving nature of this OSA-associated brain injury is suggested by the findings of our study. The coexistence of hyper- and hypotrophic changes in OSA group implies intricate and dynamic interaction of various noxius events alongside workings of the endogenous repair systems in the brain, and these may include ischemic preconditioning and enhanced neurogenesis [21,27,58].

### 4.1 Hypertrophic changes and relation to previous studies

The role for the altered neurogenesis and possible conditioning effect of OSA in our patients was suggested by the significant enlargement of hippocampal volumes of up to 4 % greater than that of the control values. Additionally, the hypertrophy of choroid plexus, an important source of adult neurogenic factors and signalling molecules for the migration of cells in the SVZ[27], was noted. These findings are compatible with reported hypertrophic change of these structures under ischaemic conditions[59]. 

Previous clinical studies of the CNS changes in OSA (as summarised in [12]) predominantly report hypotrophic changes in OSA patients. Allowing for the fact that the cross-sectional design of our study permits for association, rather than any claim of the causal relationship, we suggest some possible explanations for this divergence. Firstly, our cohort represented patients with mixed disease severity and hence potentially differed from other investigated cohorts that incorporated patients on the more severe end of the OSA spectrum[12]. Indeed, the duration of the exposure to IH and the intensity of the hypoxia bouts are important determinants of whether IH is protective or harmful[4,26,32]. Secondly, our patients were relatively young, mostly in their forties with no obvious major co-morbidities, and possibly at early stages of disease onset. The age-dependent decline in adult neurogenesis is an accepted phenomenon, although it appears to be mediated more by the age-related alterations in the cellular environment than impaired responsiveness of progenitor cells to neurogenic stimuli[29]. 

Unlike the earlier studies that utilized the optimized VBM method, this study used the fully automated FS analysis that was proven particularly effective for analysis of subcortical structures[44,49,50]. Conversely, the whole-brain VBM method has been shown in a study as less sensitive than the other methods when it comes to detecting abnormalities in small subcortical structures[49]. In a recent magnetic resonance spectroscopy study of OSA patients, decreased frontal lobe neuronal viability and integrity and decreased hippocampal membrane turnover was shown although the use of VBM method did not show any lesions in the same patients in those regions[42]. It should be noted that in animal studies, subregions of hippocampus were shown to be differentially sensitive to chronic IH[23,26,30]. For example CA1 was particularly IH-sensitive and prone to increased levels of apoptosis whilst CA3 and DG were significantly less so[23]. DG was additionally able to undergo compensatory neurogenesis[31]. Further enhancements of cognitive vulnerability to IH exposures occurred in CA1 in rats fed on an obesity-inducing diet[60]. It is, hence, possible that depending on the balance of these changes and their overall offset, the whole volume of the hippocampus might be ultimately noted as either hyper- or hypotrophic. Finally, volume increase in hippocampus could represent an epiphenomenal, or downstream, effect of the connectivity with other brain regions, which include prefrontal cortex, amygdala and thalamus. 

### 4.2 Hypotrophic changes

In the current study, hypotrophic changes were noted in OSA patients in the middle anterior portion of corpus callosum and a trend of reduced volume in the right thalamus. This is in accordance with previous clinical studies[12] and possibly supports the hypothesis that a disturbed thalamocortical oscillator underlies some of the neurodeficits[10,61]. One of the major sources of thalamic afferents to the hippocampus (e.g. CA1 and subiculum) is from nucleus reuniens, the largest of the midline nuclei of the thalamus, the region known to be strongly activated by chronic IH[62,63]. Nucleus reuniens has been implicated in associative learning and object recognition and it is proposed to gate information flow between the hippocampus and the medial prefrontal cortex[64,65]. 

Similarly, the noted reduction of the mid-anterior portion of corpus callosum in the OSA patients in our study is in agreement with previous diffusion tractography (DTI) studies of white matter tracts changes; it likely represents the effects of IH on the later myelinating part of this tract[66,67]. 

### 4.3 Correlations with volumes of cerrebellar cortex

Our group has previously shown hypotrophic changes in cerebellar cortices of OSA patients[14]. We also suggested that functional deficits noted in OSA, such as for example dysmetria of thought and affect, could be seen as the by-product of being at the milder end of spectrum of cerebellar cognitive affective syndrome [10,68]. In this study, no significant differences in cerebellar volumes were recorded although aberrant connectivity with hippocampal structures was suggested by the interregional volume correlations analysis (Figure **1B**). 

Although there are no direct monosynaptic anatomical connections between hippocampi and cerebellum, their connectivity is thought to be important for the control of movement under states of heightened emotion, novel conditions, and for the associative learning. Hippocampus is connected to cerebellum via the pontine, reticular and olivary nuclei whilst the return loop is via the fastigial nucleus and thalamus[69]. Recently, a role for hippocampal theta oscillations in coordinating a widely distributed memory system for associative learning, of which cerebellum is a part, has been proposed[70]. Moreover, it was suggested that hippocampal theta oscillations, also thought to play the role in hippocampal neurogenesis[27], can modulate the functional properties of the cerebellum[70]. 

Whilst these volumetric correlations can be only very tentatively taken to suggest a true aberrant connectivity[53] in the OSA group, they nonetheless circumstantially intimate that ‘compensatory’ entraining of cerebellum by hypertrophic hippocampi may occur. 

### 4.4 Limitations

This study did not incorporate neuropsychological testing and the lack of the related correlational data with the noted volumetric changes means that no conjecture about the compensatory role of the prominent enlargement of hippocampi can be made. Furthermore, correlations between regional volumes were exploratory and hypothesis generating and therefore should be interpreted cautiously, as well as confirmed in future studies. It should be noted that the ultrastructural determinants of group differences in morphology of the hippocampus and thalamus are unknown. Addressing these limitations will require detailed post-mortem and other in vivo (adult neurogenesis) imaging methods in order to determine those ultrastructural underpinnings. Finally, the strict exclusion criteria used in this study disallows for any judgments to be made regarding interactions between OSA and its comorbidities such as hypertension and diabetes, both strongly associated with OSA and also known to cause brain injury[2,9,71]. 

## Conclusion

In summary, our findings demonstrate for the first time the hypertrophy of hippocampus in OSA patients with mixed disease severity. It is proposed that these enlargements represent the end effect of the neuroglial ischemic preconditioning[21,22,26,29,58]. This interpretation is consistent with extensive preclinical evidence that increased hippocampal neurogenesis occurs in response to IH, which consequently increases in volume and thickness[29,31,33,72]. Aberrant connectivity between limbic regions and cerebellum was also inferred by our study. However, limitations of our study include the moderate sample size and cross-sectional design, which can suggest only an association rather than a causal relationship between noted changes in OSA patients.

It has been previously suggested that increasing age and OSA work additively (or even synchronistically) to overwhelm the brain’s capacity to respond to cognitive challenges with compensatory recruitment, and to maintain performance[1,9,13]. It would be, hence, important to recognise which compensatory mechanisms evoked by OSA are functionally viable and which may be further detrimental, especially in older people. In order to comprehensively address this problem, a detailed and multimodal mapping of discrete changes in hippocampal subregions/subfields of OSA patients with different AHI severities, matched BMIs and of different age groups will be needed.

## Supporting Information

Table S1 **Percentage Ratios of Subcortical Volumes to the ICV as determined by FreeSurfer.**
(DOCX)

Table S2 **Interregional Correlations.**
(DOCX)
