# Supplementary material for: Hippocampal Hypertrophy and Sleep Apnea: A Role for the Ischemic Preconditioning?
Source: PLoS One. 2013 Dec 13;8(12):e83173. doi: 10.1371/journal.pone.0083173 (PMC3862721; doi:10.1371/journal.pone.0083173)
Supplement: Table S1 — Percentage Ratios of Subcortical Volumes to the ICV as determined by FreeSurfer. (DOCX) [file pone.0083173.s001.docx]

**Table S1. Percentage Ratios of Subcortical Volumes to the ICV as determined by FreeSurfer.**

| Structures |  | Mean  N=32 | Std. Deviation | Student *t-test^a^* P values | ANCOVA^a^ *P* values |
| --- | --- | --- | --- | --- | --- |
| Left-Cerebellum-White-Matter | OSA | 0.972 | 0.130 | 0.852 | 0.758 |
|  | Control | 0.978 | 0.155 |  |  |
| Left-Cerebellum-Cortex | OSA | 3.348 | 0.289 | 0.459 | 0.366 |
|  | Control | 3.413 | 0.344 |  |  |
| Left-Thalamus-Proper | OSA | 0.457 | 0.046 | 0.539 | 0.302 |
|  | Control | 0.464 | 0.056 |  |  |
| Left-Caudate | OSA | 0.229 | 0.028 | 0.133 | 0.149 |
|  | Control | 0.219 | 0.026 |  |  |
| Left-Putamen | OSA | 0.373 | 0.045 | 0.266 | 0.316 |
|  | Control | 0.360 | 0.048 |  |  |
| Left-Pallidum | OSA | 0.111 | 0.012 | 0.867 | 0.761 |
|  | Control | 0.112 | 0.014 |  |  |
| Left-Hippocampus | OSA | 0.293 | 0.032 | 0.057 | 0.067 |
|  | Control | 0.278 | 0.029 |  |  |
| Left-Amygdala | OSA | 0.108 | 0.012 | 0.165 | 0.193 |
|  | Control | 0.103 | 0.015 |  |  |
| Left-choroid-plexus | OSA | 0.120 | 0.020 | 0.104 | 0.048 |
|  | Control | 0.111 | 0.021 |  |  |
| Right-Cerebellum-White-Matter | OSA | 0.964 | 0.104 | 0.411 | 0.363 |
|  | Control | 0.992 | 0.166 |  |  |
| Right-Cerebellum-Cortex | OSA | 3.450 | 0.281 | 0.495 | 0.416 |
|  | Control | 3.504 | 0.344 |  |  |
| Right-Thalamus-Proper | OSA | 0.441 | 0.038 | 0.225 | 0.094 |
|  | Control | 0.455 | 0.057 |  |  |
| Right-Caudate | OSA | 0.230 | 0.026 | 0.480 | 0.530 |
|  | Control | 0.226 | 0.023 |  |  |
| Right-Putamen | OSA | 0.353 | 0.042 | 0.241 | 0.286 |
|  | Control | 0.340 | 0.047 |  |  |
| Right-Pallidum | OSA | 0.104 | 0.012 | 0.494 | 0.563 |
|  | Control | 0.101 | 0.016 |  |  |
| Right-Hippocampus | OSA | 0.285 | 0.030 | 0.042 | 0.049 |
|  | Control | 0.269 | 0.031 |  |  |
| Right-Amygdala | OSA | 0.105 | 0.013 | 0.152 | 0.179 |
|  | Control | 0.099 | 0.015 |  |  |
| Right-choroid-plexus | OSA | 0.142 | 0.026 | 0.051 | 0.024 |
|  | Control | 0.129 | 0.026 |  |  |
| CC_Posterior | OSA | 0.060 | 0.007 | 0.580 | 0.591 |
|  | Control | 0.059 | 0.011 |  |  |
| CC_Mid_Posterior | OSA | 0.027 | 0.005 | 0.383 | 0.458 |
|  | Control | 0.026 | 0.006 |  |  |
| CC_Central | OSA | 0.027 | 0.007 | 0.505 | 0.455 |
|  | Control | 0.028 | 0.007 |  |  |
| CC_Mid_Anterior | OSA | 0.027 | 0.004 | 0.074 | 0.039 |
|  | Control | 0.030 | 0.009 |  |  |
| CC_Anterior | OSA | 0.057 | 0.007 | 0.935 | 0.978 |
|  | Control | 0.057 | 0.011 |  |  |
| SubCortGrayVol | OSA | 11.917 | 0.820 | 0.804 | 0.648 |
|  | Control | 11.974 | 1.006 |  |  |

In the table, for each neuroanatomical structure statistical analysis of group differences for volumes normalised to the ICV was performed; t-test and ANCOVA test (covariate with age), were done.

^a^Bonferroni corrected P values. *****Significant difference between OSA patients and healthy controls (P<.05).

**Abbreviations**: **ANCOVA**, Analysis of covariance; **CC**, corpus callosum; **ICV**, intracranial volume; **OSA**, obstructive sleep apnoea; **SD**, standard deviation.
