# Supplementary material for: Hippocampal Hypertrophy and Sleep Apnea: A Role for the Ischemic Preconditioning?
Source: PLoS One. 2013 Dec 13;8(12):e83173. doi: 10.1371/journal.pone.0083173 (PMC3862721; doi:10.1371/journal.pone.0083173)
Supplement: Table S2 — Interregional Correlations. (DOCX) [file pone.0083173.s002.docx]

**Table S2. Interregional Correlations.^*^**

|  | **r (P value)** | |
| --- | --- | --- |
| **Interregional Correlation** | **Control** | **OSA** |
| L CC with RH | 0.201 (.269) | 0.379 (.032) |
| L CC with LH | 0.184 (.313) | 0.357 (.045) |
| R CC with LH | 0.124 (.499) | 0.326 (.069) |
| R CC with RH | 0.168 (.359) | 0.363 (.041) |

**^*^** Pearson correlation coefficients for cerebellar cortical regions and the hippocampus in the OSA and the control groups.

**Abbreviations**: **L**, left; **R**, right; **CC**, cerebellar cortex; **H**, hippocampus; **OSA**, obstructive sleep apnoea.
